# Process Optimization for Polyphenol Extraction from Macroalgae Residues and Assessment of Their Compositions, Antioxidant Activities, and Glycosidase Inhibition

**DOI:** 10.3390/foods14173055

**Published:** 2025-08-29

**Authors:** Xianxian Luo, Hao Chen, Jiayi Mi, Xinyan Li, Ziheng Wu, Yan Jiang, Xiufang Dong

**Affiliations:** College of Public Health, Dali University, Dali 671000, China; 18577113703@163.com (X.L.); ch_chenhao_2001@163.com (H.C.); 13100092616@163.com (J.M.); 19238840950@163.com (X.L.); 13619667257@163.com (Z.W.); jiang_ya@126.com (Y.J.)

**Keywords:** macroalgae residue, bioactive compounds, antioxidant activity, enzymatic activity, bound polyphenols

## Abstract

Macroalgae are often used to produce sodium alginate, but their by-products have not been fully utilized. This study aimed to optimize the extraction of bound polyphenols (BPs) from *Macrocystis pyrifera* (L.) residues, analyze the composition of free polyphenols (FPs) and BPs, and evaluate their antioxidant activities and ability to inhibit glycosidase activity. The optimal conditions for extracting BPs achieved by the response surface method were as follows: 50 °C, a solid–liquid ratio of 1:50, an alkaline hydrolysis time of 2.38 h, and a NaOH concentration of 8 mol/L. Polyphenol content determination results indicated that FPs had significantly higher total polyphenols (13.02 ± 0.05 μg GAE/mg) and phlorotannin (3.44 ± 0.04 μg PE/mg) than BPs (6.57 ± 0.07 μg GAE/mg and 1.32 ± 0.20 μg PE/mg). HPLC/ESI-QTOF-MS showed distinct profiles: FPs had one polyhydroxy phenol, nine flavonoids, and four additional compounds, whereas BPs had five flavonoids and four other compounds. Antioxidant activity was found to be higher in FPs than in BPs (DPPH: 3.03 vs. 1.79 μg TE/mg; FRAP: 19.40 vs. 7.43 μg TE/mg). Furthermore, FPs exhibited 4.59- and 11-fold higher inhibition capacity toward α-amylase and α-glucosidase, respectively, compared to BPs. The results provide valuable basic data for the application of macroalgae residues in the marine biological industry and reveal their potential hypoglycemic ability.

## 1. Introduction

Polyphenols are the most significant group of compounds that determine the biomedical importance of marine algae [1]. Algae have been demonstrated to contain large amounts of polyphenols [2], which exhibit various bioactive properties, including antitumor [3], anti-inflammatory [4], and anti-diabetic [5] effects. These findings underscore the necessity for more comprehensive and multidimensional investigations into the polyphenolic compounds in algae. Polyphenols are divided into free polyphenols (FPs) and bound polyphenols (BPs) according to solubility and extraction methods [6]. The extraction of BPs is usually more complicated than that of FPs, often requiring chemical or enzymatic hydrolysis [7,8]. There is research showing the potential antioxidant and anti-inflammatory activities of BPs in algae, which are beneficial to human health [9]. Consequently, optimizing extraction techniques for BPs in algae warrants systematic investigation to fully exploit their bioactive potential.

Macroalgae are aquatic photosynthetic organisms that are mainly classified as green, red, or brown algae [10,11]. *Macrocystis pyrifera* (L.) Ag. is an alga of the genus *Macrocystis* under the family Laminariaceae, which is part of the order Laminariales within the phylum Heterokontophyta [12]. Macroalgae are rich in nutrients such as flavonoids, tannins, and polysaccharides, which are often used to extract sodium alginate. Sodium alginate is a macroalgae extract that exhibits significant antioxidant activity and has broad application prospects [13]. Research has shown that sodium alginate helps prevent diseases such as cardiovascular disease, diabetes, and non-alcoholic fatty liver disease (NAFLD) due to its richness in bioactive compounds like polyphenols and flavonoids [14,15]. Beyond their ecological roles, macroalgae are commercially utilized in food processing and agricultural applications [16,17]. However, studies have shown that about 30–50% of the raw materials in the macroalgae industry eventually become residues [18]. As a by-product of the extraction process of sodium alginate, macroalgae residue is mainly used for feed, fertilizers, and biological refining [19], as it is still rich in polyphenols, dietary fiber, and other active components, which have not been fully utilized. There is a significant research gap regarding the methodological optimization of macrocystis residue extraction and subsequent analysis of their biological properties.

The hyperglycemia potential of algal polyphenols has been extensively investigated [5], while studies on the bioactivity of polyphenols from macroalgae residues currently remain limited. Investigating the inhibitory activities of α-amylase and α-glucosidase from macroalgae residues will facilitate future exploration of their potential beneficial effects on diabetes management. The present study focuses on achieving the reuse of active ingredients in the macroalgae residue by optimizing the extraction of polyphenols from these residues, investigating their composition via high-performance liquid chromatography–electrospray ionization–quadrupole time-of-flight tandem mass spectrometry (HPLC-ESI-QTOF-MS/MS), and evaluating their antioxidant activity and ability to inhibit glycosidase, thereby uncovering the potential uses of macroalgae residue as a source of natural antioxidants that are suitable for functional food formulations or pharmaceutical products aimed at hyperglycemia management.

## 2. Materials and Methods

### 2.1. Materials and Chemicals

#### 2.1.1. Samples

The macroalgae residue obtained after alginate extraction was derived from *Macrocystis pyrifera* (provided by Qingdao Mingyue Seaweeds Group Co., Ltd., Qingdao, Shandong, China). The residue was dried at 55 °C, crushed into a powder by a grinder, and sieved through a 60-mesh sieve.

#### 2.1.2. Reagents and Equipment

Folin’s phenol reagent (1 mol/L), 3,5-Dinitrosalicylic acid (DNS), α-amylase (3.7 U/mg, Bacillus), and 1,1-diphenyl-2-trinitrophenylhydrazine (≥98%) were purchased from Solarbio Science & Technology Co., Ltd. (Beijing, China). Formic acid (98.0%) was purchased from Sigma-Aldrich. Gallic acid (99%), resorcinol (>99.0%), 2,4-dimethoxybenzaldehyde (DMBA) (98%), and pNPG (99%) were purchased from Macklin Biochemical Co., Ltd. (Shanghai, China). 2,4,6-Tripyridyl-s-triazine (98%) and a-glucosidase (33 U/mg, Saccharomyces) were purchased from Yuanye Bio-Technology Co., Ltd. (Shanghai, China). 4-Nitrophenyl octanoate (90%) was purchased from Aladdin Biochemical Technology Co., Ltd. (Shanghai, China). Other reagents were purchased from Sinopharm Chemical Reagent Co., Ltd. (Shanghai, China).

### 2.2. Extraction of FPs

**Figure 1:**
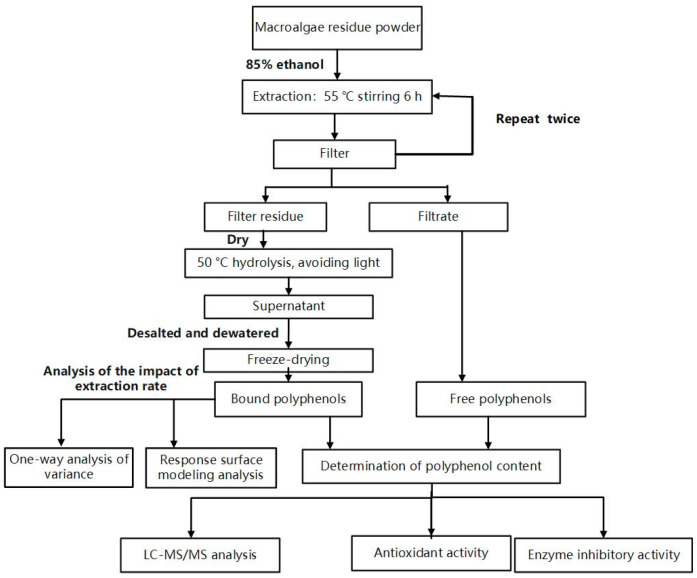
Technical flowchart.

The approach used in this study is illustrated in Figure 1. FPs were obtained via extraction with ethanol and water. The conditions for extracting free phenols from the macroalgae residue include an ethanol volume fraction of 85%, a solid–liquid ratio of 1:10, and magnetic stirring at 55 °C for 6 h. After extraction and filtration, we repeated the extraction process with ethanol twice and retained the filtrate. We then dried the filter residue at 55 °C and stored it.

### 2.3. Single-Factor Extraction Experiments for BPs

The extraction process for BPs was optimized using alkaline hydrolysis followed by ethanol precipitation. Firstly, the *Macrocystis pyrifera* residue was added to a specified volume of NaOH solution at a predetermined concentration, and the mixture was hydrolyzed at a controlled temperature. Subsequently, two volumes of 95% ethanol were added, and extraction was carried out for 1 h with continuous shaking. The extract was then centrifuged at 1118× *g* for 15 min, and the supernatant was collected for bound polyphenol quantification. Taking the rate of BP extraction as the outcome, we fixed other parameters and investigated the effects of four factors: temperature (30, 40, 50, 60, 70, 80, and 90 °C); NaOH concentration (2, 4, 6, 8, and 10 mol/L); alkaline hydrolysis time (1, 2, 3, 4, and 5 h); and liquid–solid ratio (1:10, 1:20, 1:30, 1:40, and 1:50), The specific experimental methods are shown in Table 1.

### 2.4. Optimization of Extraction Conditions and Quantification of Total Phenolic Contents

The optimal method for extracting BPs from the macroalgae residue was determined via the Box–Behnken response surface method (RSM). A three-factor and three-plane heart cube design was used, including 16 experimental runs. The NaOH concentration (A, mol/L), extraction time (B, h) and liquid–solid ratio (C) at 50 °C were altered, and the response variable was the polyphenol content (Y). The specific experimental scheme and results are shown in Table 2.

The total phenol and phlorotannin (phloroglucinol as the standard) contents of the samples extracted under the optimized conditions were determined simultaneously. The total phenol content (TPC) test method was a modified version of the Folin–Ciocalteu method, and the DMBA assay was modified from the phlorotannin content test [20]. Given the thermolability of polyphenolic antioxidant activity, single-factor tests additionally incorporated temperature effects. The experimental data was analyzed using Design Expert software (version 8.0.6).

### 2.5. Qualitative Analysis of Phenolic Compounds by HPLC-ESI-QTOF-MS/MS

Samples were dissolved in 100 μL of methanol and centrifuged at 1610× *g* and 4 °C for 10 min using a Microfuge 22R Centrifuge (Beckman Coulter, Brea, CA, USA). Then, the supernatant was extracted and used in the subsequent steps.

Capillary HPLC (Ultimate 3000, Thermo Fisher, Waltham, MA, USA) with a C18 column was used for gradient elution, with the mass spectrometer (TripleTOF5600 +, AB SCIEX, Redwood, CA, USA) in positive/negative ion mode. A Sepax GP-C18 (1.8 µm, 120Å, 2.1 mm×150 mm) column (Sepax Technologies, Newark, DE, USA) was employed for separation. The flow rate employed was 0.3 mL/min and the column was held at a constant temperature of 40 °C. The gradient elution parameters were as follows: 0~10 min with 5% B, 10~17 min with 70% B, 17~18 min with 100% B, 18~19 min with 100% B, and 19~21 min with 5% B. The ESI source was operated in positive and negative ion modes. The data were imported into MS-DIAL 4.70 software for data processing and compared with data from three libraries: MassBank, Respect, and GNPS (14,951 records in total).

### 2.6. Antioxidant Activity and Glycosidase Activity Inhibition Assay

#### 2.6.1. Antioxidant Activity Assay

The antioxidant activity of free and bound polyphenol extracts was evaluated via the DPPH and FRAP methods, respectively. The antiradical activity of DPPH was determined using an assay, as explained elsewhere [21,22]. Aliquots of 0.5 mL of Trolox standard solutions at varying concentrations were transferred to 1.5 mL microcentrifuge tubes, followed by the addition of 0.5 mL of a freshly prepared 0.2 mmol/L DPPH methanolic solution. After mixing, the reaction was carried out for 0.5 h in the dark, and absorbance was measured at 520 nm. All samples, blanks, and controls were prepared in triplicate. The radical scavenging activity of DPPH was calculated as follows:

Scavenging rate (%) = [(A_c_ − A_n_ + A_b_)/A_c_] × 100% (1)

The FRAP method was modified from Rumpf et al.’s work [23]. A total of 0.05 mL of the Trolox gradient solution was added to 1.45 mL of the FRAP working solution. The reaction was carried out in a water bath at 37 °C for 10 min, and absorbance was measured at 593 nm with a microplate reader. All samples, blanks, and reference substances were prepared in parallel and in triplicate. From the Trolox calibration curve, the results of the antioxidant activity assays were expressed as the concentration in Trolox equivalent (TE), and the calculation formula was as follows:

TE = (A_n_/A_T_) × C_T_ (2)

#### 2.6.2. Glycosidase Activity Inhibition Assay

Both FPs and BPs extracts inhibited the activities of α-glucosidase and α-amylase The method for determining the inhibitory activity of α-amylase was modified from Liu et al.’s study [24]. The diluted free and bound polyphenol extracts were added to different tubes with either 0.05 mL of the α-amylase solution (0.1 U/mL), 0.1 M pH 6.8 PBS, or 0.05 mL of an inhibitor, and then shaken at 37 °C for 15 min. Then, 0.1 mL of a starch solution (1%) was added to each tube and the reaction was carried out at 50 °C for 10 min. Each tube was supplemented with 0.4 mL of a DNS solution in a boiling water bath for 10 min, and then cooled to room temperature. Then, 1 mL of water was added to each tube, and the absorbance at 540 nm was measured. All samples, blanks, and reference substances were prepared in parallel and in triplicate. The inhibition rate was calculated using Formula (3) [25].

Inhibition rate (%) = [1 − (OD_A_ − OD_a_)/(OD_B_ − OD_b_)] × 100 (3)

The methodology for assessing the inhibitory activity of α-glucosidase has been described by Yang et al. [26]. First, we added 0.1 M phosphate buffer, an inhibitor, and α-glucosidase to the experimental group, the control group, and the blank group according to the protocol. The mixture was incubated in a water bath at 37 °C for 10 min. Subsequently, 0.1 mL of pNPG (5 mmol/L) was added to each group, and the samples were incubated at 37 °C for 1 h. Then, 1 mL of a 1 mol/L sodium carbonate solution was added. All samples, blanks, and controls were prepared in parallel and in triplicate. Absorbance was measured at 405 nm. The formula for calculating the inhibition rate is shown in Formula (3).

### 2.7. Statistical Analysis

Three separate extraction experiments were performed to determine the results, which are presented as the mean ± standard deviation. SPSS 16.0 software was used to analyze the data, and one-way ANOVA followed by the LSD (least significant difference) mean test was applied. A *p*-value of *p* < 0.05 was considered statistically significant.

## 3. Results and Discussion

### 3.1. Quantification of Free Polyphenols from the Macroalgae Residue

Through extraction and assays, it was determined that the crude extract yield of FPs from the macroalgae residue was 4.5%. The polyphenol content was 13.02 ± 0.07 μg GAE/mg and 3.45 ± 0.04 μg PE/mg. In contrast, the rate of BP extraction varied under different experimental conditions.

### 3.2. Optimized Conditions for Bound Polyphenols

**Figure 2:**
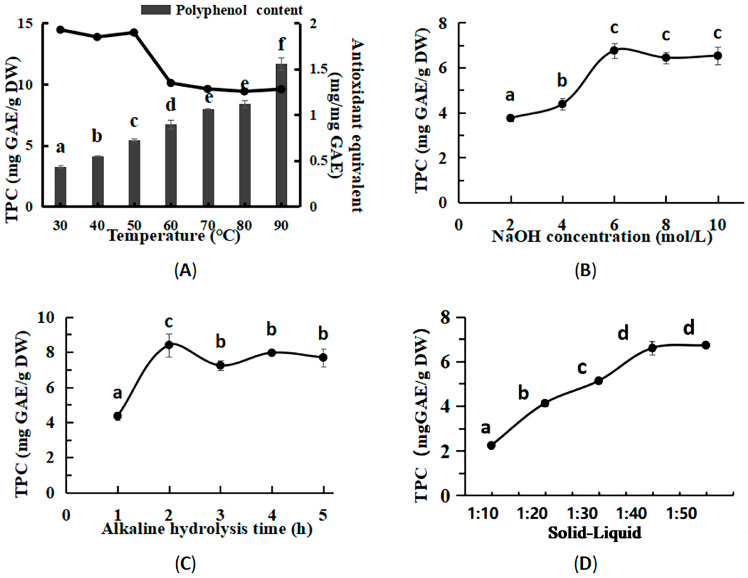
The effect of various factors on BP extraction from the macroalgae residue. The effects of temperature on BP content and antioxidant equivalents extracted from macroalgae residue through alkaline hydrolysis (4 mol/L NaOH, 1 h, and a 1:40 solid–liquid ratio). (**A**) The effects of NaOH concentration on BP content extracted from the macroalgae residue through alkaline hydrolysis (1 h, 50 °C, and a 1:40 solid–liquid ratio). (**B**) The effects of extract time on BP content extracted from the macroalgae residue through alkaline hydrolysis (4 mol/L NaOH, 50 °C, and a 1:40 solid–liquid ratio). (**C**) The effects of the solid–liquid ratio on BP content extracted from the macroalgae residue through alkaline hydrolysis (4 mol/L NaOH, 50 °C, and 1 h). (**D**). Note: Different superscript characters reveal the significant differences (*p* < 0.05).

In the single-factor experiments, the influence of various factors on the extraction rate and antioxidant activity of BPs from macroalgae residue was evaluated. The design and results of the Box–Behnken experiments are shown in Table 3. As shown in Figure 2A, the efficiency of BP extraction exhibited a positive correlation with the alkaline hydrolysis temperature from 30 °C to 90 °C. This enhancement is attributed to increased molecular mobility and accelerated solvent diffusion at higher temperatures, which reduce viscosity and improve the solubility and extraction yield of polyphenols [27]. The extract maintained high antioxidant activity within the temperature range of 30 °C to 50 °C, beyond which a continuous decline was observed from 60 °C to 90 °C. The optimal extraction effect was observed at an alkali hydrolysis temperature of 50 °C. Additionally, according to Figure 2B–D, the optimal extraction conditions for BPs were a solid–liquid ratio of 1:40, a NaOH concentration of 6 mol/L, and an alkaline hydrolysis time of 2 h.

**Figure 3:**
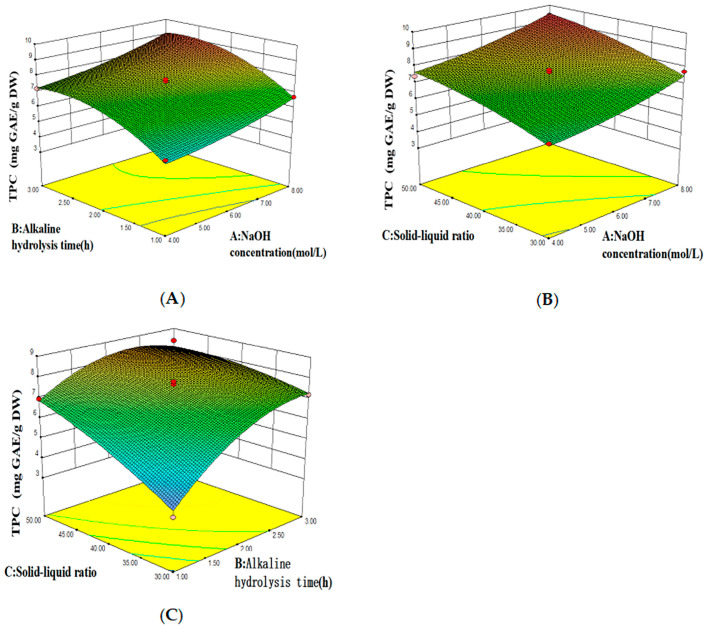
Response surface plot of variables and their mutual interactions. Contour plots of cross-interaction between NaOH concentration and (**A**) alkaline hydrolysis time and (**B**) the solid–liquid ratio and (**C**) between the alkaline hydrolysis time and the solid–liquid ratio.

As evidenced by the ANOVA results presented in Table 4, the established model demonstrates statistical significance (*p* < 0.05), confirming its validity. The significance of the factors on the response value was determined to decrease in the order C > B > A. The lack-of-fit test yielded a *p*-value of 0.0547, indicating that the lack-of-fit error is negligible compared to the pure error. The regression model, representing the amount of BPs extracted from macroalgae residues as a function of the NaOH concentration (Figure 3A), extraction time (B), and solid-to-liquid ratio (C), is given by the following equation:

Y = −9.75 − 0.47A + 6.63B − 0.39C + 0.035AB − (3.21119E − 003) AC − 0.05541BC + 0.058A^2^ − 0.87B^2^ − (2.57172E − 003) C^2^ (4)

The steepness of the slope in the response surface 3D plot reflects the strength of the interaction between the two factors; a steeper slope indicates a stronger influence on the response value, while a gentler slope suggests a weaker effect [28]. Four parallel experiments were carried out. The optimal extraction rate was achieved with a solid–liquid ratio of 1:50, alkaline hydrolysis time of 2.38 h and a NaOH concentration of 8 mol/L at 50 °C. The purity of BPs was 9.34 μg GAE/mg, and 97.19% of the predicted value could be achieved.

After extracting FPs, BPs were extracted under the optimal extraction conditions. The BP content was 6.51 ± 0.45 μg GAE/mg and 1.32 ± 0.20 μg PE/mg.

### 3.3. Characterization of the Two Polyphenol Extracts

#### Characterization of Polyphenol Extracts by HPLC-ESI-QTOF-MS/MS and Comparison with MSn Data from an Online Database

**Figure 4:**
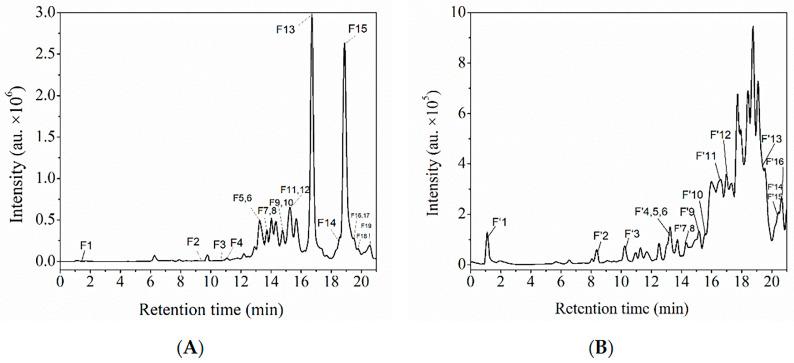
HPLC chromatograms of FPs from the macroalgae residue in the positive (**A**) and negative (**B**) ion modes.

**Figure 5:**
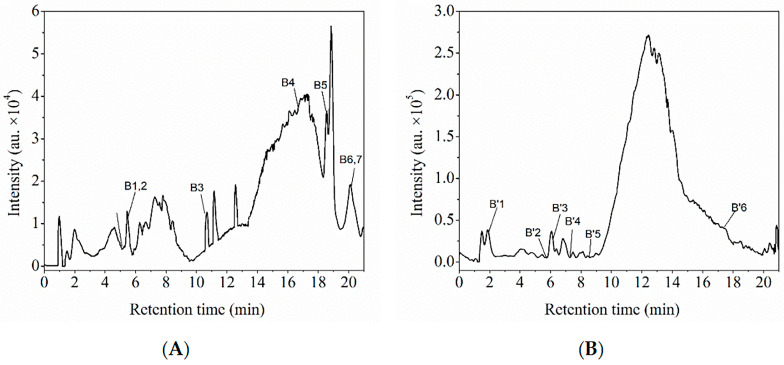
HPLC chromatograms of BPs from the macroalgae residue in the positive (**A**) and negative (**B**) ion modes.

The total ion chromatograms (TICs) of FPs and BPs, obtained from the HPLC analysis, are shown in Figure 4 and Figure 5, respectively. The TIC profiles display well-resolved peaks, indicating the presence of multiple compounds in the sample. FPs were predominantly ionized in negative mode, whereas BPs were ionized in both positive and negative modes.

In this study, FPs and BPs from the macroalgae residue were analyzed using HPLC-ESI-QTOF-MS/MS and the retention times (Rt), formulae, and accurate mass spectral data were compared with online databases and the literature (Table 5). The FPs contained 14 identifiable compounds, including 1 polyhydroxy phenol, 9 flavonoids, and 4 other phenolic substances. In contrast, the BPs comprised nine compounds, consisting of five flavonoids and four other phenolic components. FPs contain more flavonoids and other substances than BPs. This difference may be attributed to the decomposition of some heat-sensitive flavonoids caused by the alkaline hydrolysis conditions in the BP extraction process [29]. The phlorotannin components in FPs and BPs were identified by matching data from the literature (Table 6). Fuhalols and eckols were identified in both FPs and BPs, but more phlorotannin species were detected in FPs. These compounds are easily detected in positive ion mode, while phenolic acids are more easily detected in negative ion mode. It may be that phlorethols were not detected in BPs because the polyhydroxy phenol group in the BPs formed an ether bond with the cell wall polysaccharide, shielding the ionization site and resulting in blocked negative ionization [30]. A comparison with published mass spectrometry data on fucoidan polyphenols showed that the polyphenols extracted from macroalgae residues were most similar to those found in *Sargassum fusiforme*, *Sargassum muticum*, *Laminaria digitata*, and *Fucus vesiculosus*.

### 3.4. Antioxidant Activity and Inhibitory Effect on Glycosidase Activity

Table 7 shows the differences in antioxidant capacity and glycosidase inhibition between the two polyphenols. The DPPH scavenging activity assay on the macroalgae residue revealed that the antioxidant capacity in FPs was higher (3.03 μg TE/mg) than that in BPs (1.79 μg TE/mg). The FRAP test results showed a consistent trend (FPs: 1.49 μg TE/mg vs. BPs: 1.14 μg TE/mg) in that the macroalgae residue’s antioxidant capacity is higher than that of the macroalgae *edulis* (0.66 ± 0.02 μg TE/mg) [31]. The high content of phenolic compounds and flavonoids was considered to be an important cause of antioxidant capacity in previous studies [32]. Therefore, FPs may exhibit better FRAP antioxidant capacity due to their high contents of flavonoid species and unique polyhydroxy phenols. The DPPH assay measures the free radical scavenging ability using the electron-providing ability, while the FRAP assay evaluates the reducing ability via electron transfer [33].

The inhibitory activity of polyphenols against α-amylase and α-glucosidase is closely related to their structural properties [34]. Experimental results revealed that FPs showed a higher capacity of glycosidase activity inhibition (α-amylase inhibition rate: 47.22%, 0.11 mg AE/mg; α-glucosidase inhibition rate: 82.85%, 21.53 μg AE/g) than BPs (α-amylase activity inhibited by 32.88%, 0.01 mg AE/mg; α-glucosidase inhibition rate: 44.71%**,** 0.99 μg AE/g). The α-glucosidase inhibition rate of FPs extracted from the macroalgae residue was higher than that of the *Nannochloropsis oculata* extract (α-glucosidase inhibition rate: 80.42%) [35]. Studies have shown that FPs’ ability to inhibit glycosidase activity is stronger than that of BPs, which is consistent with the results of this experiment [36]. Notably, the disparity in glycosidase inhibitory activity between FPs and BPs was markedly greater than that observed for their antioxidant activities, and this phenomenon may arise from the difference in hypoglycemic activity of key differential constituents between these two polyphenols [8].

## 4. Conclusions

The optimized alkaline hydrolysis conditions led to a high BP extraction efficiency, demonstrating the effectiveness of this method for liberating cell-wall-bound bioactive compounds. A total of 23 polyphenols were identified, with 14 species for FPs and 9 species for BPs. This compositional divergence aligns with previous reports on algal polyphenol profiles, in which flavonoids dominated the free fraction. FPs exhibited greater abundance, and they contained one additional compound, potentially leading to a superior antioxidant ability and a higher glycosidase inhibition efficacy in FPs‌. There was a strong positive correlation between TPC, antioxidant activity, and glycosidase inhibition ability. This study verified macroalgae residues as a sustainable source of bioactive polyphenols, providing a preliminary understanding of their hypoglycemic activity and emphasizing their value as underutilized resources. Future research should focus on the quantitative determination of flavonoids and on elucidating the mechanism of the hypoglycemic effect and other biological activities.

## Figures and Tables

**Table 1:** Single-factor experimental scheme.

No.	Liquid–Solid Ratio	NaOH Concentration (mol/L)	Alkaline Hydrolysis Time (h)	Alkaline Hydrolysis Temperature (°C)
1	1:40	4	1	30, 40, 50, 60, 70, 80, 90
2	1:40	2, 4, 6, 8, 10	1	50
3	1:40	4	1, 2, 3, 4, 5	50
4	1:10, 1:20, 1:30, 1:40, 1:50	4	2	50

**Table 2:** Factors and levels of the RSM.

Level	A: NaOH Concentration (mol/L)	B: Alkaline Hydrolysis (h)	C: Liquid–Solid Ratio
−1	4	1	1:30
0	6	2	1:40
1	8	3	1:50

**Table 3:** The design and results of the Box–Behnken experiments.

No.	NaOH Concentration	Alkaline Hydrolysis Time (B)	Liquid–Solid Ratio (C)	Total Polyphenol Content (Y)
1	4 (−1)	1 (−1)	40 (0)	5.27
2	8 (1)	1 (−1)	40 (0)	6.69
3	4 (−1)	3 (1)	40 (0)	7.18
4	8 (1)	3 (1)	40 (0)	8.88
5	4 (−1)	2 (0)	30 (−1)	5.98
6	8 (1)	2 (0)	30 (−1)	7.72
7	4 (−1)	2 (0)	50 (1)	7.38
8	8 (1)	2 (0)	50 (1)	9.38
9	6 (0)	1 (−1)	30 (−1)	3.57
10	6 (0)	3 (1)	30 (−1)	7.20
11	6 (0)	1 (−1)	50 (1)	6.94
12	6 (0)	3 (1)	50 (1)	8.35
13	6 (0)	2 (0)	40 (0)	7.49
14	6 (0)	2 (0)	40 (0)	7.67
15	6 (0)	2 (0)	40 (0)	7.80
16	6 (0)	2 (0)	40 (0)	7.60

**Table 4:** Variance analysis of the model.

Source	Sum of Squares	Degree of Freedom	Mean Square	F-Value	*p*-Value	Significant
Model	28.27	9	3.14	39.54	0.0001	**
A	5.87	1	5.87	73.86	0.0001	**
B	10.44	1	10.44	131.48	<0.0001	**
C	7.19	1	7.19	90.54	<0.0001	**
AB	0.019	1	0.019	0.24	0.6409	
AC	0.016	1	0.016	0.21	0.6646	
BC	1.23	1	1.23	15.47	0.0077	**
A^2^	0.22	1	0.22	2.73	0.1496	*
B^2^	3.02	1	3.02	37.98	0.0008	**
C^2^	0.26	1	0.26	3.33	0.1178	
Residual	0.48	6	0.079			
Lack of fit	0.43	3	0.14	8.67	0.0547	
Pure error	0.049	3	0.016			
Cor. total	28.74	15				

Note: ** indicates *p* < 0.01, which means a highly significant difference; * indicates *p* < 0.05, which means a significant difference.

**Table 5:** Characterization of polyphenol extracts via HPLC-ESI-QTOF-MS/MS and comparison with MSn data from an online database.

Identity	Formula	Retention Time (min)	Addition Ion	*m*/*z*	Reference (*m*/*z*)	Peak
Free Polyphenols						
Polyhydroxybenzene						
5-{8(Z),11(Z)-pentadecadienyl}resorcinol	C_21_H_32_O_2_	14.0939	[M−H]^−^	315.3153	315.3175	F′8
Flavonoids						
6,8-dihydroxy-2,2,4,4-tetramethyl-7-(3-methylbutanoyl)-9-(2-methylpropyl)-9H-xanthene-1,3-dione	C_26_H_34_O_6_	20.41	[M−H]^−^	441.3863	441.3867	F′15
5-O-methylgenistein	C_16_H_12_O_5_	16.68	[M−H]^−^	283.3203	283.3212	F′11
Epiafzelechin (2R,3R)(-)	C_15_H_14_O_5_	9.148	[M−H]^−^	273.1768	273.1747	F′2
5-hydroxy-2′,4′,7,8-tetramethoxyflavone	C_19_H_18_O_7_	20.18	[M−H]^−^	339.3171	339.3141	F′14
7,4′-dimethoxysoflavone	C_17_H_14_O_4_	20.65	[M−H]^−^	281.3031	281.2998	F′16
Catechin	C_15_H_14_O_6_	1.25	[M−H]^−^	289.127	289.1219	F′1
3-[(Z)-heptadec-10-enyl]benzene-1,2-diol	C_23_H_38_O_2_	15.65	[M−H]^−^	345.2745	345.2799	F′10
Neobavaisoflavone	C_20_H_18_O_4_	10.64	[M+H]^+^	345.1133	345.11	F3
2′′-O-rhamnosyl icariside II	C_33_H_40_O_14_	15.4	[M+H]^+^	683.2292	683.23	F12
Others						
Fraxin	C_16_H_18_O_10_	14.82	[M+H]^+^	409.0553	409.0532	F10
α-Tochopherol	C_29_H_50_O_2_	15.33	[M−H]^−^	429.3738	429.3738	F′9
Esculin	C_15_H_16_O_9_	1.4	[M+H]^+^	363.0636	363.06	F1
2-hydroxy-6-pentadecylbenzoic acid	C_22_H_36_O_3_	13.53	[M−H]^−^	347.2638	347.258	F′6
Bound polyphenols						
Flavonoids						
2′,5′-dihydroxy-4-methoxychalcone	C_16_H_14_O_4_	8.52	[M−H]^−^	269.1664	269.1674	B′5
5-O-methylgenistein	C_16_H_12_O_5_	17.35	[M−H]^−^	283.3191	283.3212	B′6
Quercetin 3,7-dimethyl ether	C_17_H_14_O_7_	1.88	[M−H]^−^	329.0576	329.062	B′7
Licoflavone A	C_20_H_18_O_4_	10.69	[M+Na]^+^	345.1138	345.11	B3
Licoricidin	C_26_H_32_O_5_	20.79	[M+H]^+^	447.2125	447.21	B7
Others						
Juglone	C_10_H_6_O_3_	5.03	[M+H]^+^	212.9939	212.9949	B1
Alpha-estradiol	C_18_H_24_O_2_	5.58	[M+H]^+^	255.1714	255.1744	B2
3-methoxy-2-(3-methylbut-2-enyl)-5-pentylphenol	C_17_H_26_O_2_	6.15	[M−H]^−^	261.1752	261.1783	B′3
Atalaphylline	C_23_H_25_NO_4_	7.28	[M−H]^−^	378.2395	378.2336	B′4

**Table 6:** Characterization of polyphenol extracts via by HPLC-ESI-QTOF-MS/MS and comparison with MSn data from the literature.

Identity	Addition Ion	Measured Mass (*m*/*z)*	MS/MS Fragment Detected (*m*/*z*)	Algae Source	Peak
Free polyphenols					
Fuhalols					
2-(1)	[M+H]^+^	267.2	141	*Sargassum fusiforme*	F5
3-(1)	[M+H]^+^	391.3	125	*Sargassum fusiforme*	F6
5-(1)	[M+H]^+^	639.3	388	*Sargassum fusiforme*	F17
5-(2)	[M+H]^+^	655.3	527, 389	*Sargassum fusiforme*	F8
6-(3)	[M+H]^+^	795.3	667	*Sargassum fusiforme*	F14
Dihydroxytetrafuhalol	[M+H]^+^	547.4	529	*Sargassum muticum*	F11
Dihydroxypentafuhalol	[M+H]^+^	671.3	625, 623, 607, 527, 465, 402, 341, and 263	*Sargassum muticum*	F4
Dihydroxyhexafuhalol	[M+H]^+^	795.3	777, 749, 731, 653, and 465	*Sargassum muticum*	F14
Pentafuhalol	[M+H]^+^	639.3	621, 513, 385, and 373	*Sargassum muticum*	F15
Hydroxytetrafuhalol	[M+H]^+^	655.3	637, 527, and 389	*Sargassum muticum*	F8
Bifuhalol trimer	[M+H]^+^	795.3	515	*Carpophyllum flexuosum*	F14
Octafuhalol	[M+H]^+^	1011.7	621	*Sargassum muticum*	F7
nd	[M+H]^+^	653.3	607 and 465	*Laminaria digitata*	F16
nd	[M+H]^+^	653.3	607 and 465	*Laminaria digitata*	F18
Dihydroxypentafuhalol	[M−H]^−^	669.6	625	*Sargassum muticum*	F′13
Dihydroxyhexafuhalol	[M−H]^−^	793.6	465	*Sargassum muticum*	F′12
Hydroxytrifuhalol	[M−H]^−^	405.2	154	*Carpophyllum flexuosum*	F′3
Eckols					
3-(1)	[M+H]^+^	389.2	329, 245, and 123	*Sargassum fusiforme*	F2
Eckol	[M−H]^−^	371.2	335	*Sargassum fusiforme*	F′7
Eckol derivative	[M−H]^−^	401.3	371	*Fucus vesiculosus*	F′5
Others					
Tetramer	[M+H]^+^	499.4	481 and 233	*Fucus vesiculosus*	F9
Fucol	[M+H]^+^	623.3	495, 479, 461, 373, and 355	*Laminaria digitata*	F13
Pentaphlorethol	[M+H]+	623.3	603 and 493	*Sargassum muticum*	F19
Pentamer	[M+H]^+^	623.3	605, 497, 434, and 356	*Fucus vesiculosus*	F13
Phlorethol	[M−H]^−^	745.5	477	*Laminaria digitata*	F′4
Bound polyphenols					
Fuhalols					
3-(1)	[M+H]^+^	391.3	123	*Sargassum fusiforme*	B5
6-(3)	[M+H]^+^	795.5	667 and 389	*Sargassum fusiforme*	B6
Dihydroxyhexafuhalol	[M+H]^+^	795.5	777, 749, 731, 513, 511, 485, 483, and 385	*Sargassum muticum*	B6
Trifuhalol	[M+H]^+^	391.3	251	*Sargassum muticum*	B5
Bifuhalol trimer	[M+H]^+^	795.5	515 and 261	*Carpophyllum flexuosum*	B6
Eckols					
6-(3)	[M+H]^+^	793.5	747 and 385	*Sargassum fusiforme*	B4
2-(1)	[M−H]^−^	263.2	245 and 219	*Sargassum fusiforme*	B′2
Others					
Fucol	[M+H]^+^	623.3	477 and 371	*Fucus vesiculosus*	B4
Fucophlorethol	[M+H]^+^	623.3	461, 373, and 355	*Fucus vesiculosus*	B4
Pentaphlorethol	[M+H]^+^	623.3	493	*Sargassum muticum*	B4
5	[M+H]^+^	623.3	605, 373, 356, 355, and 340	*Sargassum muticum*	B4

**Table 7:** Comparison chart of antioxidant and enzyme inhibitory activity.

Polyphenol Type	DPPH (μg TE/mg)	FRAP (μg TE/mg)	Anti-Amylase (mg AE/mg)	Anti-Glucosidase (μg AE/g)
Free	3.03	19.40	0.11	21.53
Bound	1.79	7.43	0.01	0.99

## Data Availability

The original contributions presented in this study are included in the article. Further inquiries can be directed to the corresponding author.
